# Organic Boundary Location Based on Color-Texture of Visual Perception in Wireless Capsule Endoscopy Video

**DOI:** 10.1155/2018/3090341

**Published:** 2018-01-10

**Authors:** Chengliang Wang, Zhuo Luo, Xiaoqi Liu, Jianying Bai, Guobin Liao

**Affiliations:** ^1^College of Computer Science, Chongqing University, Chongqing 400044, China; ^2^Department of Gastroenterology, Second Affiliated Hospital, Third Military Medical University, Chongqing, China

## Abstract

This paper addresses the problem of automatically locating the boundary between the stomach and the small intestine (the pylorus) in wireless capsule endoscopy (WCE) video. For efficient image segmentation, the color-saliency region detection (CSD) method is developed for obtaining the potentially valid region of the frame (VROF). To improve the accuracy of locating the pylorus, we design the *Monitor-Judge* model. On the one hand, the color-texture fusion feature of visual perception (CTVP) is constructed by grey level cooccurrence matrix (GLCM) feature from the maximum moments of the phase congruency covariance and hue-saturation histogram feature in HSI color space. On the other hand, support vector machine (SVM) classifier with the CTVP feature is utilized to locate the pylorus. The experimental results on 30 real WCE videos demonstrate that the proposed location method outperforms the related valuable techniques.

## 1. Introduction

**Figure 1:**
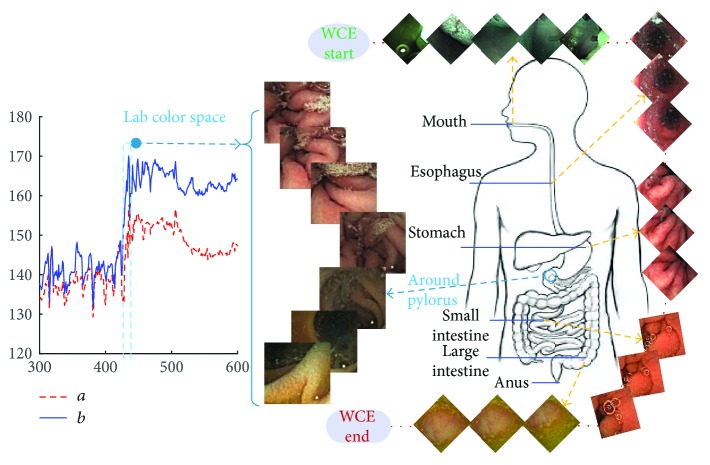
WCE videos about different organs of the digestive system and color change curve in Lab color space around the pylorus. WCE videos are provided by Jinshan. And some sample frames of a video show the results of screening digestive system by WCE. The pylorus is the end of the stomach and the boundary between the stomach and the small intestine, where the color of the images has changed markedly as shown in the *a/b* color curve. After images have been converted into Lab color space, the *a/b* color curve is generated by the average values in channel *a* and *b* of the continuous images around the pylorus.

Wireless capsule endoscopy (WCE) was invented by a group of researchers in Baltimore in 1989 and introduced by Given Imaging Inc. as a commercial tool [1]. And it has a good performance in screening bleeding, ulceration, submucosal swelling, worms, polyps, and cancer, which is a vital breakthrough in the comprehensive examination of gastrointestinal tract (GI) which is painless [2, 3]. So far, the WCE system of Chongqing Jinshan Science and Technology Group (Jinshan) mainly consists of the camera capsule, image recorder, capsule guiding device, and image workstation [4]. The capsule enters the digestive tract from the mouth and captures the images of the digestive tract by a digital camera in the capsule. However, a WCE video generally lasts over 8 hours and contains more than 40,000 frames which bring the clinician a heavy burden for the diagnosis and treatment of diseases. Even an experienced clinician will take over an hour at least to analyze the data of each patient on average. Therefore, it is necessary to detect lesion images automatically [2]. But different digestive tract organs have different textures, which means that the same lesion appears differently in different organs as shown in Figure 1. For this reason, the computer researchers [5, 6] generally find the organic boundaries for segmenting the WCE video according to different organs before recognizing lesion images in a single organ. Yuan and Meng [5] and Karargyris and Bourbakis [6] just detect the lesion images in the small intestine, but they both manually select the part of WCE video about the small intestine for avoiding the disturbance of other organs. However, it is a time-consuming and laborious task to locate the organic boundaries artificially. So it is important to automatically locate organic boundaries, which not only can help the clinician to ensure relevant organ section and reduce the review time, but also is the vital preliminary work for automatic lesion recognition [7]. In this paper, we propose a novel method to locate the boundary between the stomach and the small intestine (the pylorus).

Many works are for WCE video segmentation and have been devoted to locate the pylorus because the key of WCE video segmentation is finding the organic boundaries. Although the researches on this work are not mature enough, they have yielded a great number of positive results. Cunha et al. [7] utilize MPEG-7 scalable color and SVM classifier to segment a WCE video into four parts including the entrance, stomach, small intestine, and large intestine based on Gaussian prior probabilities. In terms of their global model fitting step, this step is a time-consuming procedure for estimating and judging all frames in a WCE video. Some other classifiers based on color have been proposed to locate the pylorus. Berens et al. [8] report a stomach/intestine classifier with the hue-saturation histograms to predict the pylorus. However, this method just achieves an average performance of 86% and 85% for accuracy and recall, respectively. Li et al. [9] use color histogram in Lab color space and textural features in wavelet domain to represent the visual content. Furthermore, they apply motion analysis approaches to segment WCE video [10]. But the best average performance of CE video segmentation in terms of recall is 71.89%. Especially, the researchers all propose a two-level approach for WCE video segmentation [11, 12]. They all firstly find the approximate positions of organic boundary and then refine the boundary. However, these two approaches are both time-consuming tasks because they need to compute almost all the images about the stomach and the small intestine in WCE videos. Although Zhou et al. [11] considers the influence of the impurities, gastric juice, and illumination, they only use some fixed thresholds that weaken the robustness of denoting the valid regions of frames. And at the rough level, he computes a completed average dissimilarity curve to find the probable boundary, which adds some computing burden. Zhou et al. [12] applies the trained KNN classifier to the improved WLD features of the images around the candidates and selects three best candidates as the output in the end. However, this method needs manual intervention to select the best one from the three candidate positions of the pylorus.

All of these above methods have ignored many interfering factors that lead to the dissatisfied accuracy and are time-consuming because they must deal with almost all the frames of the WCE video. The proposed method in this paper successfully shortens the time of locating the pylorus and improves the location accuracy by contrast with some of the above methods.

The contributions in this paper can be summarized into the following three points:

1. The CSD method is proposed to obtain the VROF region effectively and adaptively to remove the bad effects of the disturbances including food debris, strong shadows, overexposure, air bubbles, and gastric juice.
2. The *Monitor-Judge* model is designed to locate the organic boundary (the pylorus) for reducing time consumption.
3. The CTVP feature is constructed by grey level cooccurrence matrix (GLCM) feature from the maximum moments of the phase congruency covariance and hue-saturation histogram feature in HSI color space, which is better to express the difference between stomach images and small intestine images than other selected features [11, 12].

## 2. Materials and Methods

In this section, a novel method is proposed to locate the pylorus in this paper. Firstly, WCE video images are divided into small windows that form a window pair sequence, and we obtain the VROF region of images by the CSD method. Secondly, we propose the *Monitor-Judge* model for locating the pylorus: *Monitor* constantly monitors the suspicious window pair with the pylorus according to the ratio of the color dissimilarity of current window pair and the average color dissimilarity of previous window pairs; *Judge* classifies images and estimates the pylorus position in the suspicious window pair by SVM classifier with the CTVP feature.

### 2.1. VROF Region Extraction

Actually, many possible disturbances such as gastric juice, shadows, excessive bright regions, and air bubbles show various appearances in different images and make it difficult to extract color or texture feature from the histology of digestive tract and therefore cause these extracted features unreliable.

**Figure 2:**
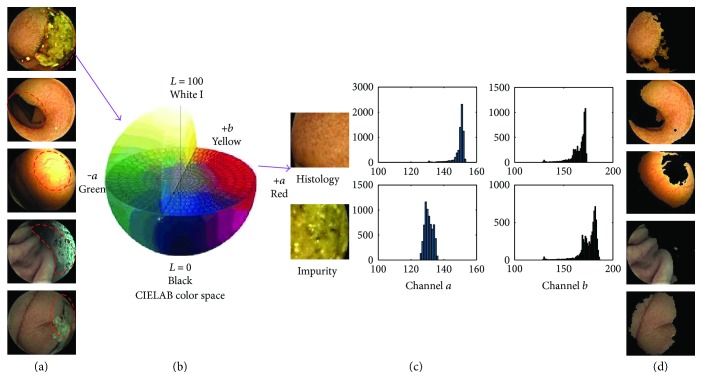
Process results of obtaining the VROF region and histogram comparison in Lab color space between digestive tract histology and the disturbance. The descriptions of the figure from left to right now follow. (a) Samples with five categories of the disturbances marked by red dashed line that are food debris, strong shadows, excessive bright region, air bubbles, and gastric juice consecutively. (b) Lab color model. (c) The comparison result of histograms between digestive tract histology and the disturbance in channels *a* and *b* in Lab domain. (d) Images processed by CSD method. The black regions are invalid and the colored parts are used for feature extraction.

The disturbances are as follows. (1) Food debris: food debris is one of the common impurities that obscure digestive tract tissue; (2) strong shadows: strong shadows are the lack of describing the real color and texture of digestive tract tissue; (3) overexposure: overexposure is one kind of image distortion caused by fierce reflection; (4) air bubbles: air bubbles are mainly caused by gastrointestinal peristalsis and pressure change; (5) and gastric juice: gastric juice is liquid commonly found in the stomach.  Figure 2(a) gives some examples of WCE images with disturbances.

Valid region of the frame (VROF) is the region of a WCE image without any disturbance. VROF can show the color and texture of digestive tract tissue clearly.

CIE Lab color is designed to approximate human vision, which aspires to perceptual uniformity [13]. The three coordinates of CIELAB represent the lightness of the color (*L*^∗^ = 0 yields black and *L*^∗^ = 100 indicates diffuse white; specular white may be higher), its position between red/magenta and green (*a*^∗^, negative values indicate green while positive values indicate magenta), and its position between yellow and blue (*b*^∗^, negative values indicate blue and positive values indicate yellow). For removing these disturbances, we convert WCE images into Lab color space and find that tissue of digestive tract shows commonly stronger color reflection in channel *a* than the color reflection in any disturbance, and the tissue has obvious difference in channel *b* with any disturbance.  Figure 2(c) shows the difference in histograms of channel *a* and channel *b* between digestive tract tissue and the disturbance. In terms of Lab color, digestive tract tissue in the image is salient region in contrast with disturbance region.

IG [14] as one of the saliency detection methods is simple to implement and computationally efficient. For obtaining the VROF region (salient region) in WCE images, the color-saliency detection (CSD) method is proposed in this paper based on IG. In the remainder of this subsection, details about the CSD method are described.

DoG filter, as a kind of band pass filters, is widely used for edge detection because it approximates the Laplacian of Gaussian (LoG) filter but it is much faster to compute than the LoG filter [14]. It satisfies detecting intensity changes when the standard deviations of the Gaussians are in the ratio 1 : 1.6 [14, 15]. DoG filter is chosen for WCE image reprocessing in the CSD method. The DoG filter is given by the following:

(1) $$\begin{array}{c} DoG\left(x,y\right)=\frac{1}{2\pi }\left[\frac{1}{\delta_{1}^{2}}e^{-\left(x^{2}+y^{2}\right)/2\delta_{1}^{2}}-\frac{1}{\delta_{2}^{2}}e^{-\left(x^{2}+y^{2}\right)/2\delta_{2}^{2}}\right]. \end{array}$$

Our method of finding the saliency map *S* for an image of width *W* and height *H* pixels is formulated as follows:

(2) $$\begin{array}{c} S\left(x,y\right)={\left(a_{u}-a\left(x,y\right)\right)}^{\alpha }+{\left(b_{u}-b\left(x,y\right)\right)}^{\beta }, \end{array}$$

where *a*_*u*_ and *b*_*u*_ are the arithmetic mean pixel values of channels *a* and *b* in Lab color space. *a*(*x*, *y*) and *b*(*x*, *y*) are the corresponding image pixel values in the Gaussian blurred results of the original image in Lab color space and *α* is greater than one and must be an odd number. When *α* is greater, the saliency region represents the area with stronger value in channel *a*. *β*  should be greater than one and smaller than  *α*. The experiments show that *α* = 3 and  *β* = 1.5 contribute to good results presented in Figure 2(d).

VROF is the region of a frame where *S*(*x*, *y*) > 0 and the results are presented in Figure 2(d).

### 2.2. *Monitor*-*Judge* Model for Pyloric Position

#### 2.2.1. *Monitor*-*Judge* Model

**Figure 3:**
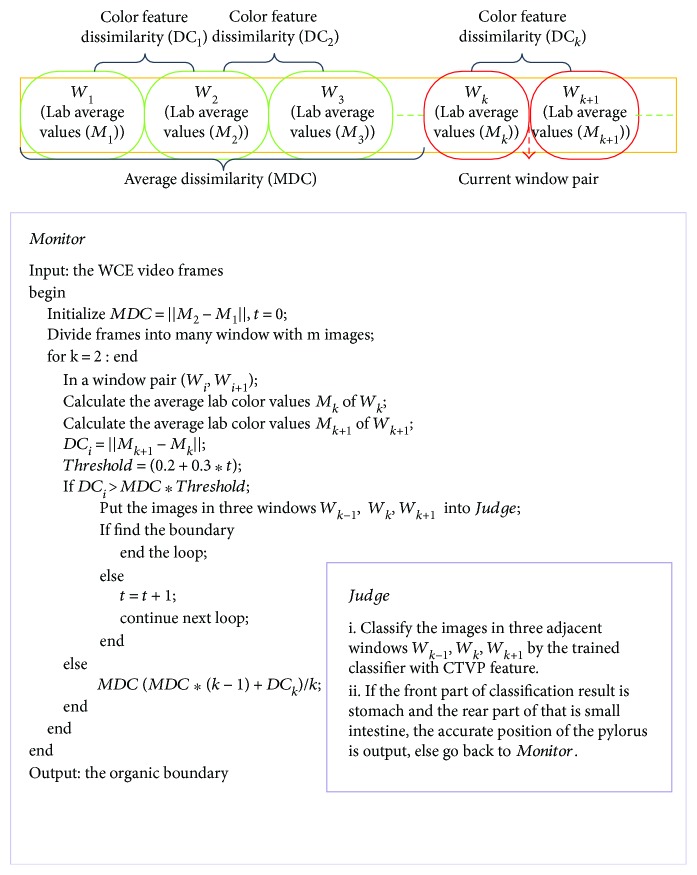
*Monitor-Judge* model. The procedures are described simply as follows. (1) Initialize *t* = 0, MDC = DC_1_; (2) compute the average Lab color values *M*_*k*_ and *M*_*k*+1_ in the current window pair (*W*_*k*_, *W*_*k*+1_); (3) compute the color dissimilarity DC_*k*_ in the window pair; (4) if DC_*k*_/MDC > 0.2 + 0.3^∗^*t*, *Judge* checks the current window pair and *t* = *t* + 1, or go back to (2) to monitor the next window pair.

In WCE videos with good quality, the sequence frames have an obvious color change when a capsule enters the next digestive organ. These characteristics can be found from the *a*/*b* color curve in Figure 1. Therefore, we design *Monitor-Judge* model for monitoring and judging the suspicious window pair with the pylorus as shown in Figure 3. For efficiency, we can divide the WCE video into many small windows with *m* images, which form window pair sequences (*W*_1_, *W*_2_), (*W*_2_, *W*_3_),…, (*W*_*n*−1_, *W*_*n*_). In this method, images are converted into Lab color space. In order to reduce the influence of luminance, only the average values of data in channels *a* and *b* of the VROF region are considered as colored features in this procedure.

(3) $$\begin{array}{c} {avg}_{c}=\frac{\sum_{i,j}I_{c}\left(i,j\right)}{N_{valid}},\\ M_{c,k}=\frac{\sum_{1}^{m}{avg}_{c}}{m}, \end{array}$$

where avg_*c*_ and *M*_*c*,*k*_ represent the average color values of a frame and the image window *k* respectively;  *c* presents any channel of Lab color space, that is, *L*, *a*, or *b*. *I*_*c*_(*i*, *j*) is the value of the pixel in the valid regions, and *N*_valid_ represents the total number of pixels in the valid region of a frame. Then, Euclidean distance is utilized to demonstrate the color dissimilarity of a window pair.

(4) $$\begin{array}{c} {DC}_{k}=\sqrt{{\left(M_{a,k}-M_{a,k+1}\right)}^{2}+{\left(M_{b,k}-M_{b,k+1}\right)}^{2}},\\ MDC=\frac{\sum_{t=1}^{t=k-2}{DC}_{t}}{k-2}, \end{array}$$

where DC_*k*_ stands for the color dissimilarity in (*W*_*k*_, *W*_*k*+1_). MDC stands for values in the average dissimilarity previous window pairs.

By analyzing many WCE videos, we find that the color of the WCE images about the same digestive organ generally have little change and the color of WCE images around the organic boundary has changed markedly. According to this, the key idea of the *Monitor-Judge* model is constantly comparing the color dissimilarity (DC_*k*_) of the current window pair *k* with the average color dissimilarity (MDC) of all previous window pairs and then considering detecting organic boundary in the current window pair. Obviously, the proposed model does not need to deal with these images after finding organic boundary and it is more efficient than the two-level approaches in [11, 12]. The experiment shows that it just needs 2.55 times of *Monitor-Judge* operation on average to find the organic boundary.

In this model, the time complexity is *O*(*m* · *W* · *H*) which is better than the method in [11]; *m* is the size of window and *W* and *H* are the width and height of an image, respectively. The pylorus appears in 400th~6000th images in our data. The intestinal peristalsis makes the capsule move forward slowly. The camera in the endoscopy takes three pictures per second, so that at least five continuous images are exactly similar. To improve efficiency, an interval of five frames in a window with 100 frames for extracting color feature will greatly reduce the cost of time through many experiments.

#### 2.2.2. The Color-Texture Fusion Feature of Visual Perception (CTVP)

**Figure 4:**
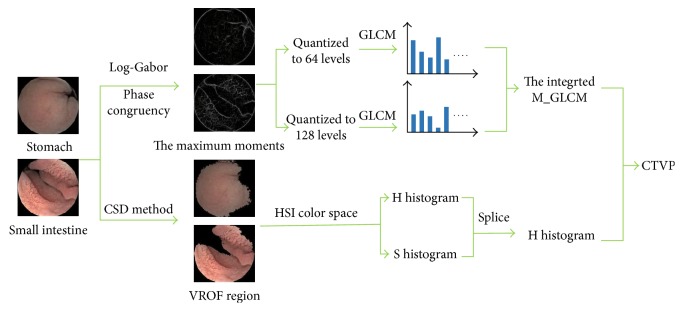
Construction of the fusion feature (CTVP) for describing the WCE image effectively.

Because there are some changes of color and texture between two adjacent organs in general, a classifier with the CTVP feature is applied to a target window pair shown in Figure 4.

**Figure 5:**
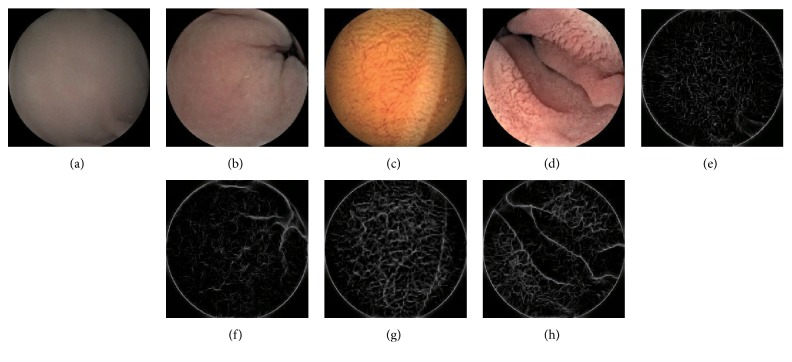
Comparison of the maximum moments of the phase congruency covariance between the stomach and the small intestine.

The small intestine has a large number of small intestine villi as texture by contrast with the stomach, and the maximum moments can highlight the textures shown in Figure 5. To extract a useful texture feature, we firstly calculate the maximum moments of phase congruency [16] based on Fourier components of original image deal with 2D log-Gabor filter [17], and then extract the integrated GLCM features from the maximum moments.

The 2D phase congruency PC_2D_(*x*) is defined as follows:

(5) $$\begin{array}{c} {PC}_{2D}\left(x\right)=\frac{\sum_{S}\sum_{O}W_{o}\left(x\right)\left\lfloor A_{so}\left(x\right)\left[\cos\left(\varphi_{so}\left(x\right)-\overset{¯}{\varphi_{o}\left(x\right)}\right)-\sin\left(\varphi_{so}\left(x\right)-\overset{¯}{\varphi_{o}\left(x\right)}\right)\right]-T_{o}\right\rfloor }{\sum_{S}\sum_{O}A_{so}\left(x\right)+\varepsilon }, \end{array}$$

where the numerator is the weighted and noise compensated local energy summed over all orientations, and the denominator is the total sum of filter response amplitudes over all orientations and scales [16]. *x* is the pixel location in the spatial domain. *W*_*o*_(*x*) is weighing function of phase congruency by frequency spread at orientation *o*.  *A*_*so*_(*x*) denotes the amplitude of the grey scale WCE image. *φ*_*so*_(*x*) denotes the phase response of original image at scale *s* and orientation *o* of log-Gabor filter. $\overset{¯}{\varphi_{o}\left(x\right)}$ represents the mean phase angle at orientation *o*. *T*_*o*_ is the estimated noise energy at orientation *o*. *ε* is small constant which prevents division by zero. ⌊ ⌋ symbol denote that the enclosed quantity is equal to itself when its value is positive and zero otherwise. An overall measure of phase congruency in the two-dimensional (2D) local energy is firstly calculated in several orientations (typically six) by using data from oriented 2D Log-Gabor wavelets [17].

The maximum moments of the phase congruency covariance is given by

(6) $$\begin{array}{c} M=\frac{\left(a+c+\sqrt{b^{2}+{\left(a-c\right)}^{2}}\right)}{2}, \end{array}$$

where *a* = ∑(PC_2D_(*o*)cos(*o*))^2^,  *b* = ∑PC_2D_(*o*)^2^sin(*o*)cos(*o*), and  *a* = ∑(PC_2D_(*o*)sin(*o*))^2^. PC_2D_(*o*) refers to the phase congruency value determined at orientation *o* and the sum is performed over the discrete set of orientations.

After getting the map of maximum moment, the cooccurrence probabilities are calculated from this map as texture features. These probabilities represent the conditional joint probabilities of all pairwise combinations of grey levels in the spatial window of interest given by two parameters: inter pixel distance (*δ*) and orientation (*θ*) [18]. The probability measure can be defined as follows:

(7) $$\begin{array}{c} C_{i,j}=\frac{P_{i,j}\left(\delta ,\theta \right)}{\sum_{i,j}^{G}P_{i,j}\left(\delta ,\theta \right)}, \end{array}$$

where *P*_*i*,*j*_(*δ*, *θ*) represents the number of occurrences of grey level pair (*i*, *j*) within the given window, given a certain (*δ*, *θ*)  pair, and *G*_*i*,*j*_ is the quantized number of grey levels. In this paper, we consider four properties as features concluding *contrast*, *correlation*, *energy*, and *homogeneity* that are formulated as follows:

(8) $$\begin{array}{c} Contrast=\sum C_{i,j}{\left(i-j\right)}^{2},\\ Correlation=\sum \frac{\left(i-u_{x}\right)\left(j-u_{y}\right)C_{i,j}}{\sigma_{x}\sigma_{y}},\\ Energy=\sum {C_{i,j}}^{2},\\ Homogeneity=\sum \frac{\left(i-u_{x}\right)\left(j-u_{y}\right)C_{i,j}}{1+\left|i-j\right|}. \end{array}$$

By analyzing many WCE images in the stomach and the small intestine, it is found that color of images between the stomach and the intestine has obvious discrimination in general. It is reported that color histogram contributes to good performance of described images among different digestive organs [11]. HSI color space decomposes an image into components of hue (H), saturation (S), and intensity (I) [19]. The intensity of image is instable because of constant movement of capsule endoscopy. So, we also choose HS histogram to represent the color features. Importantly, after RGB images are converted into HSI space, we calculate the HS histogram from the VROF region.

(9) $$\begin{array}{c} {Hist}_{H}\left(i\right)=\frac{n_{H}\left(i\right)}{N_{valid}},\\ {Hist}_{S}\left(i\right)=\frac{n_{S}\left(i\right)}{N_{valid}}, \end{array}$$

where *N*_valid_ is the total number of pixels in the VROF region, *n*_*H*_(*i*) and *n*_*S*_(*i*) are the frequency of the *i*th bin in H and I channels of the VROF region, and *i* = 1, 2,…, 16.

SVM classifier [20] with RBF kernel is utilized for classifying images in three target windows. Then we judge the position of the pylorus from the classification results. In this step, the time complexity from GLCM is *O*(*ηN*) where *η*  is the range of the intensity level (e.g., 256).

## 3. Results and Discussion

### 3.1. Dataset and Experiment Design

The WCE video data used in these experiments is acquired from different patients with different ages and provided by Jinshan in Chongqing. There are 30 videos being used for the experiments in this paper, and each video contains more than 42000 frames with 256 × 240 pixels. It is worth mentioning that our samples are very diverse and come from all over the world like China, Middle East, and Europe. For privacy reasons, the names of the cases are not real and just indicate where they are from. We randomly select 3801 images around the pylorus in eight videos, and 1822 are before the pylorus, 1979 are after the pylorus. That is to say, there are 3801 frames included in the training and validation set. The label of the images that before and after the pylorus are 0 and 1, respectively. In addition, another nine cases are used for testing the accuracy of the estimate position of the pylorus based on the proposed method in this paper. The rest of the videos are for demonstrating the importance of the VROF region.

The accuracy of locating the pylorus is assessed by the error frames between the boundary obtained from the experiments and the one manually labelled by three clinicians. The mean and the median errors are considered in experiment results. The mean error is the average error of all test videos and the median error is the middle error value in all test videos. To verify the effectiveness of our proposed algorithms, three traditional performance metrics such as accuracy, sensitivity (recall), and specificity are measured in our experiments. Those three performance metrics are described as follows:

(10) $$\begin{array}{c} Accuracy=\frac{P_{T}+N_{T}}{P_{T}+P_{F}+N_{T}+N_{F}},\\ Sensitivity=\frac{N_{T}}{P_{F}+N_{T}},\\ Specificity=\frac{P_{T}}{N_{F}+P_{T}}, \end{array}$$

where *P*_T_ is the number of actually positive frames predicted as positive frames, *N*_T_ is the number of actually negative frames predicted as negative frames, *P*_F_ is the number of true positive frames predicted as negative frames, and *N*_F_ is the number of true negative frames predicted as positive frames.

To demonstrate the operation of the proposed method, we perform six sets of experiments.

The first experiment confirms the importance of segmenting the VROF region. The second one compares the performance of SVM classifier, K-nearest neighbour (KNN) classifier, and Naive Bayes classifier. SVM classifier with RBF kernel is selected in this experiment. The parameter *K* used in KNN classifier is 5. The third one compares the CTVP feature with HS histogram, the ULBP feature, and the improved WLD. The fourth one evaluates the influence of different window sizes on locating the pylorus. The fifth one compares and analyzes the performance of different methods. The last one gives analysis of the location error of the proposed method.

In order to obtain convincing results, all the systems run on our own data. On the one hand, our results of the proposed method are compared with the existing methods which we try our best to reimplement based on the literature. On the other hand, these results are also compared with the ground truth (GT); GT has been generated artificially by three clinicians who are from Third Military Medical University, China. For convincing research, the error standard is set up by those three clinicians.

### 3.2. Experiment Results

The first experiment is for revealing the importance of segmenting the VROF region.  Table 1 shows the efficiency of finding right window with the pylorus based on entire frames and VROF regions by frequency and time consumption. Frequency is smaller, the efficiency of finding the right window with the pylorus is better. Based on the VROF region, it just needs 2.55 times of the *Monitor-Judge* operation and about 16 seconds to find the right window with the pylorus. The results clearly confirm the significance of valid region denotation. And the better performance of using VROF regions can be explained by the fact that it is necessary to reduce the negative influence brought by gastric juice, shadows, excessive bright regions, and bubbles.

**Figure 6:**
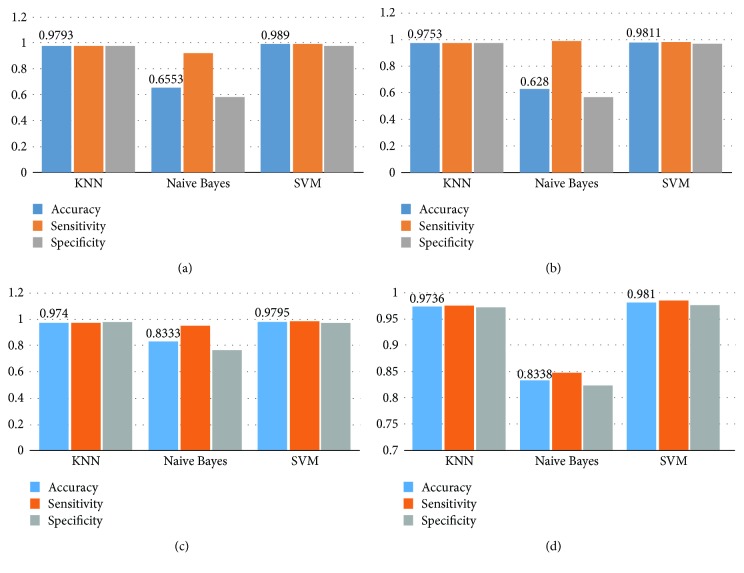
Classification performance of different classifiers and the influence of different parameters of the CSD method on the CTVP feature. (a) The CSD method with *α* = 3, *β* = 1.5. (b) The CSD method with *α* = 5, *β* = 3. (c) The CSD method with *α* = 7, *β* = 5. (d) CSD method with *α* = 9, *β* = 7.

The second experiment is for evaluating the classification performance of different classifier and selecting the appropriate classifier in the proposed method. As shown in Figure 6, we apply 10-fold cross validation operations 10 times to evaluate the classification performance of KNN classifier, Naive Bayes classifier, and SVM classifier based on the different values of *α* and *β* in the CSD method. It is found that the CTVP feature is reliable and effective to describe the WCE images of different digestive organ, because SVM classifier and KNN classifier both obtains the acceptable classification results. From Figure 6(a), *α* = 3 and  *β* = 1.5 contribute to the best performance of KNN classifier and SVM classifier; and the average accuracy of SVM classifier are 98.9%, which has an increase of 1% than that of KNN classifier. Because a peculiarity of the KNN classifier is that it is sensitive to the local structure of the training data, the SVM classifier with RBF kernel is a more appropriate choice for the proposed method.

The third experiment is for presenting a comparison between different features. The results in Table 2 are acquired by 10-fold cross validation method to display the performance of different features including HS histogram, the ULBP feature, the improved WLD, the integrated M_GLCM, and the CTVP feature. It is found that the proposed the CTVP feature is more reliable and effective for classifying the stomach and the small intestine than these comparison features. The improved WLD is not good choice to describe the WCE images in our training data because the average accuracy of SVM classifier with the improved WLD is just 86.0%. The average accuracy of the CTVP feature are 98.9%, which is more suitable to describe an image than independent color or texture feature. HS histogram and ULBP [11] achieves an average performance of 97.1% and 95.4% for accuracy and specificity, respectively. Nevertheless, it is not more excellent than the CTVP feature in this paper.

**Figure 7:**
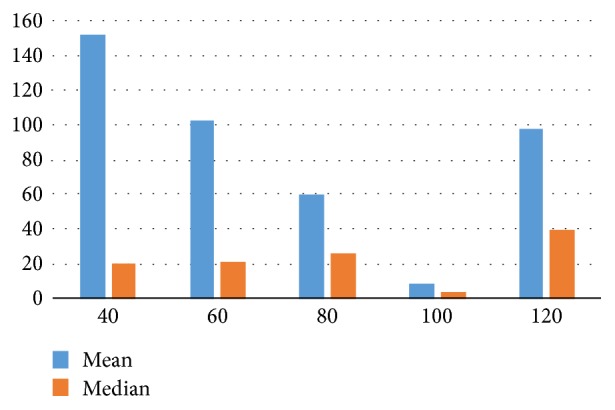
Mean error and median error of locating the pylorus in the proposed method with different sizes of sliding window. The *x*-axis represents the different sizes (*m* in Section 2.2.1) of sliding window and the *y*-axis shows the location error.

The fourth experiment is for evaluating the influence of different window sizes on locating the pylorus. In Figure 7, we evaluate the influence of different values of *m* on locating the pylorus by mean error and median error. The results shows that the proposed method with *m* = 100 has the best performance of locating the pylorus. This be explained by the fact that *m* = 100 can weaken the influence of classification error in our data set.

**Figure 8:**
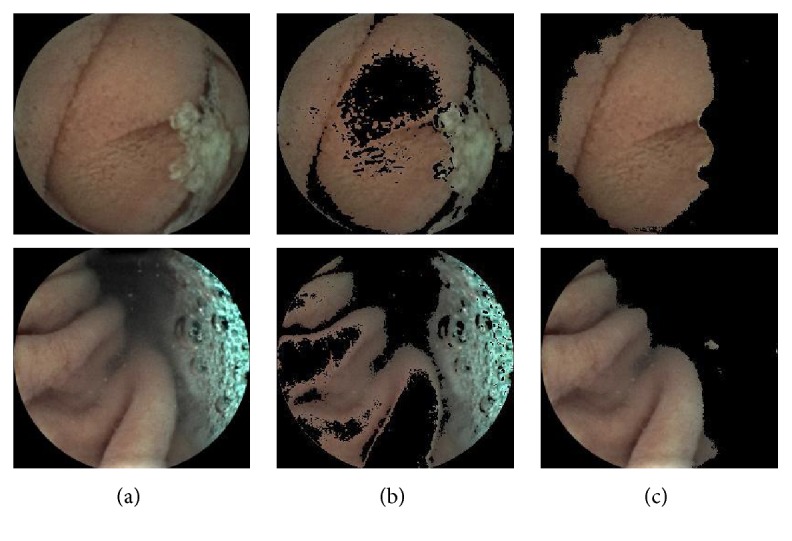
Different method for extracting the VROF region. (a) Original images with gastric juice and bubbles. (b) The process results of (a) based on Zhou R.'s operation [11]. (c) The process results of (a) based on CSD method.

The fifth experiment is for comparing the performance of the proposed method with two existing valuable methods. In Table 3, we compare and analyze the performance of different location methods by the location accuracy and the time consumption. Zhou et al. [12] get three candidate positions of the pylorus as the output. Table 3 just shows the best one of three candidates of the pylorus based on Zhou S.'s approach. The error of Zhou S.'s approach is 360 and 201 frames of mean and median, respectively. It is because Zhou et al. [12] do not think about the disturbances in images, which makes a bad influence on the selection of the candidates of the pylorus and leads to poor performance of classification. Although Zhou et al. [11] has considered the negative effects of the disturbances that cause the result of extracting features unreliable, it is lack of robustness to denote valid regions with some fixed condition in HSI color space. The examples in Figure 8 show that the proposed CSD method is more adaptive to extract the VROF region than Zhou R.'s method. Zhou R. uses the peak of the completed dissimilarity curve in Lab color space to locating the pylorus approximately. However, not only computing this completed dissimilarity curve is time-consuming, but also the position of the pylorus may not appear near the peak in our data because of the shortage of his mechanism for denoting the valid regions in WCE images. If they fail in the rough level, it is impossible to find the correct position of the pylorus in the fine level. These defects of Zhou R.'s method lead to the low accuracy (mean error is 1385 frames) and long execution time (141 minutes). The *Monitor-Judge* model shows excellent performance on locating the pylorus in WCE video: On the one hand, the error of locating the pylorus is just 9 and 4 frames of mean and median, respectively; on the other hand, the proposed method takes less time (1.26 minutes on average) to locating the pylorus than these two exiting methods.

**Figure 9:**
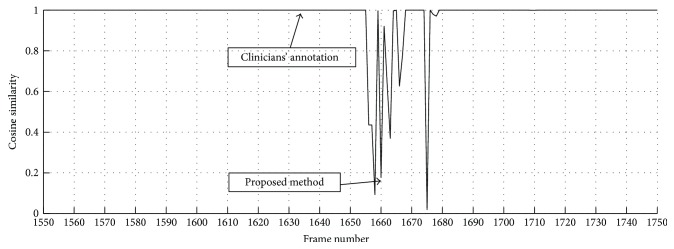
The curve of cosine similarity based on H histogram in HSI color space. The color features are extracted from1551~1750 frames in the case—Feng. The 1634th fame is annotated as the pylorus by three clinicians and 1660th frame is the result of the proposed method.

The sixth experiment is for analyzing the reason of the location error. As shown in Table 3, there is an obvious distinction of the position of the pylorus between clinician and our method in Feng. In Figure 9, we analyze the location error in the video (Feng) by the cosine similarity of color feature. The result shows that the color features of the frames around the position of the pylorus that annotated by clinician do not have a distinct change in general. However, the position that annotated by the proposed method is clearly marked boundary of the color feature. Due to some hardware problems or other unknown factors, this error is caused by the color distortion of the image that also makes trouble for the clinicians. In consideration of the importance of color feature for describing the stomach and the small intestine, the error is generally acceptable by the clinicians.

## 4. Conclusions

In this paper, we have introduced an effective method to locate the pylorus in WCE videos. Firstly, the CSD method is designed to obtain the VROF region for the color feature extraction, which can remove the bad effects of the disturbances including food debris, strong shadows, overexposure, air bubbles, and gastric juice. Secondly, the *Monitor*-*Judge* model and the CTVP feature are proposed to promote the efficiency and accuracy of locating the pylorus. Based on color variation rule of sequential images, the proposed *Monitor*-*Judge* model reduces the time consumption of locating the pylorus. And the CTVP feature is very suitable to describe the images about the stomach and the small intestine. Thirdly, the SVM classifier is applied to predict frames in the target windows. And the position of the pylorus in WCE video is determined by analyzing the classification errors of the SVM classifier. Experimental results show that the proposed approach outperforms the techniques proposed in [11, 12] in terms of the location efficiency and accuracy on a database of 30 WCE videos. In the future, we will collect more WCE videos to verify the validity of the method for locating the boundary between the small intestine and the large intestine and investigate new methods for abnormality detection in different digestive organs.

## Figures and Tables

**Table 1:** Performance comparison of using entire frame and valid region.

	Frequency				Time consumption (s)
	Mean	Median	Max	Min	Average
Entire region	3.59	1.5	19	1	161
VROF region	2.55	1.5	7	1	16

Frequency: the times of monitoring needed to find the right window with the pylorus. Time consumption: time consumption for finding the right window with the pylorus.

**Table 2:** Classification rates of different features.

Features	Accuracy	Sensitivity	Specificity
HS histogram and ULBP [11]	97.1%	98.7%	95.4%
Improved WLD [12]	86.0%	90.2%	82.5%
The integrated M_GLCM	94.09%	94.68%	93.51%
HS histogram (VROF region)	93.44%	96.13%	90.86%
CTVP	98.9%	99.7%	98.1%

**Table 3:** The results of the different location methods.

Cases	Clinicians' annotation	Zhou R.'s method [11]		Zhou S.'s method [12]		The proposed method	
	Position	Position	Time (min)	Position	Time (min)	Position	Time (min)
Duan	4849	5061	≈141	4861	3.89	4852	2.43
Liu	5440	4082		3921	2.88	5420	2.81
Feng	1634	4790		1271	3.41	1660	1.31
Wang	2042	1202		1991	1.77	2044	1.6
AMTA	710	578		501	2.86	708	0.56
AMM	1380	1192		1331	2.87	1380	0.37
RG	4019	1355		3531	2.33	4032	0.58
NNY	1704	4239		1511	3.23	1708	1.44
*Mean*	—	1385	141	360	2.91	9	1.39
*Median*	—	1099	141	201	2.78	4	1.26

*Mean* is the average error of locating the pylorus and *median* is the median of errors in the testing cases. *Time* represents time needed for locating the pylorus.
